# Meropenem for children with severe pneumonia: Protocol for a randomized controlled trial

**DOI:** 10.3389/fphar.2022.1021661

**Published:** 2022-11-17

**Authors:** Xue Tian, Lei Dong, Ting-Ting Jiang, Bo-Hao Tang, Ze-Ming Wang, Yue-E Wu, Dian-Ping You, Jing Bi, Su-Yun Qian, Hui Qi, A-Dong Shen

**Affiliations:** ^1^ Laboratory of Respiratory Diseases, Beijing Key Laboratory of Pediatric Respiratory Infection Diseases, Beijing Pediatric Research Institute, Beijing Children’s Hospital, Capital Medical University, Key Laboratory of Major Diseases in Children, Ministry of Education, National Clinical Research Center for Respiratory Diseases, National Center for Children’s Health, Beijing, China; ^2^ Department of Pharmacy, Children’s Hospital of Hebei Province, Shijiazhuang, Hebei, China; ^3^ Baoding Key Laboratory of Precision Diagnosis and Treatment of Children Infectious Diseases, Baoding Children’s Hospital, Baoding, Hebei, China; ^4^ Key Laboratory of Chemical Biology, Ministry of Education, Department of Clinical Pharmacy, School of Pharmaceutical Sciences, Cheeloo College of Medicine, Shandong University, Jinan, China; ^5^ Department of Pediatrics, Beijing Friendship Hospital, Capital Medical University, Beijing, China; ^6^ Pediatric Research Institute, Children’s Hospital of Hebei Province, Shijiazhuang, Hebei, China; ^7^ Pediatric Intensive Care Unit, Beijing Children’s Hospital, National Center for Children’s Health, Capital Medical University, Beijing, China

**Keywords:** meropenem, severe pneumonia, children, randomized controlled trial, study protocol

## Abstract

**Background:** Pneumonia, caused by infection or other factors, seriously endangers the health of children. Meropenem is an effective broad-spectrum antibiotic using in the treatment of infectious diseases. In the therapy of pneumonia, meropenem is mostly employed for the treatment of moderate to severe pneumonia. Previously, we established a population pharmacokinetics (PPK) model for meropenem in pediatric severe infection and simulated the control rate of the time during which the free plasma concentration of meropenem exceeds the minimum inhibitory concentration (MIC) is 70% of the dosing interval (70% fT > MIC). Therefore, we plan to conduct a multicenter randomized controlled trial (RCT) to compare the efficacy and safety between conventional regimen and model regimen for meropenem in pediatric severe pneumonia.

**Methods:** One hundred patients (aged 3 months to 15 years) will be recruited in this RCT. They will be assigned randomly (at a 1:1 ratio) to a conventional treatment group (20 mg/kg, q8h, with 0.5–1 h infusion) and a model treatment group (20 mg/kg, q8 h, with 4 h infusion). The primary outcome will be 70% fT > MIC. Secondary outcomes will be the prevalence of meropenem therapy failure, duration of antibiotic therapy, changes in levels of inflammatory indicators, changes in imaging examination results, and prevalence of adverse events. Ethical approval of our clinical trial has been granted by the ethics committee of Beijing Children’s Hospital ([2022]-E-133-Y). This trial has been registered in the Chinese Clinical Trial Registry (ChiCTR2200061207).

**Discussion:** Based on our previous PPK data, we have designed this RCT. It is hoped that it will promote rational use of antibacterial drugs in children suffering from severe pneumonia.

**Clinical Trial Registration**: http://www.chictr.org.cn identifier, ChiCTR2200061207.

## Introduction

Pneumonia is a serious disease threatening the health of children; 800,000 children die because of pneumonia each year worldwide (Unicef, 2020). Severe pneumonia seriously affects the health of children, but also leaves debilitating sequelae. Accordingly, severe pneumonia elicits heavy burdens to families and society worldwide.

*Streptococcus pneumoniae* (S. *pneumoniae*) and *Haemophilus* *influenzae* (H. influenzae) are more frequently associated with pneumonia in children. (Li et al., 2021). Meropenem is a carbapenem antibiotic and is one of the important antibiotics in the treatment of severe pneumonia (National Health Commission and Medicine, 2019). Studies have shown that the clinical cure rate of intermittent infusion (II) is significantly lower than that of extended infusion (EI) (Lorente et al., 2006), and its control rate of plasma concentration is lower (Kongthavonsakul et al., 2016; Hassan et al., 2020). However, EI will significantly improve meropenem plasma concentration (Zhou et al., 2021; Lan et al., 2022). Therefore, to improve the therapeutic effect and reduce the risk of antibiotic resistance, carrying out studies on the population pharmacokinetics (PPK) and pharmacodynamics (PD) of meropenem in treatment of severe pneumonia in children is a rational approach.

To assess the PK features of meropenem in children, we undertook a PPK study of meropenem in children suffering from a severe bacterial infection. We established a PPK model and discovered that bodyweight and the rate of creatinine clearance were significant covariates affecting the clearance of meropenem in children. In addition, prolonging the duration of intravenous infusion could significantly increase the control rate of the time during which the free plasma concentration of meropenem exceeds the minimum inhibitory concentration (MIC) is 70% of the dosing interval (70% fT > MIC) (Wang et al., 2020). Based on those data, we plan to conduct a multicenter randomized controlled trial (RCT) on the treatment effect of meropenem in children with severe pneumonia. The duration of meropenem treatment will be decided by clinicians according to patients’ symptoms, physical examination and related auxiliary examinations. We will compare the control rate of 70%fT > MIC between patients in model regimen and conventional group, and the MIC will be decided by the nationwide epidemiological data. Then, we will evaluate their efficacy and safety by secondary outcomes. Accordingly, we hope to provide a theoretical basis for anti-infective therapy in children with severe pneumonia.

## Methods

### Randomized controlled trial design

**FIGURE 1:**
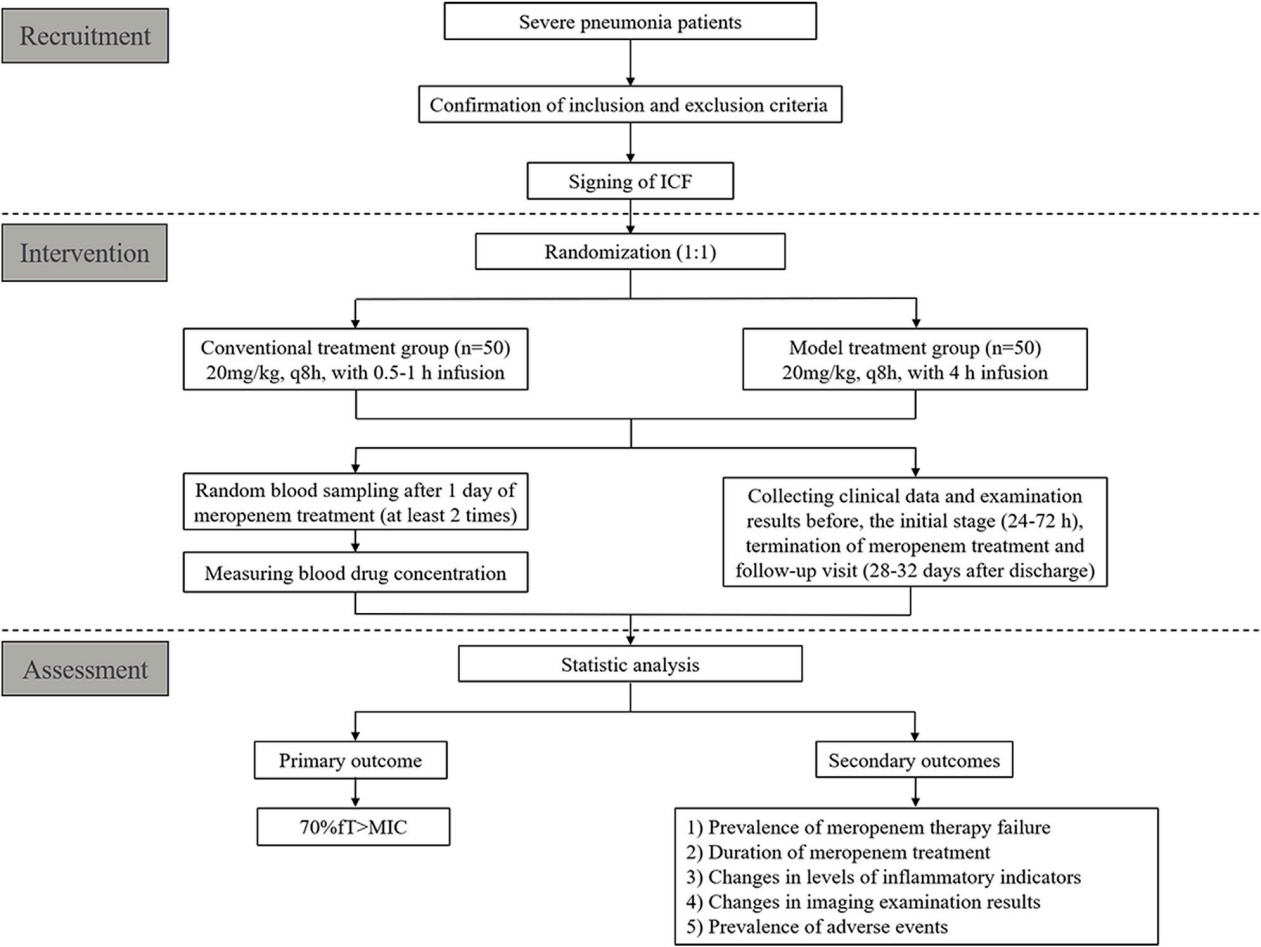
The schematic diagram of the RCT design.

This will be a multicenter, single-blind RCT for children with severe pneumonia treated with meropenem. We plan to recruit 100 patients from four hospitals in China from September 2022 to September 2023 (Table 1). These patients will be assigned randomly to one of two groups at a 1:1 ratio: conventional treatment group and model treatment group. A schematic diagram of RCT design is shown in Figure 1. The details of patient enrollment, study interventions, and outcome assessments are shown in Table 2. This RCT design is referred to the Consolidated Standards of Reporting Trials (CONSORT) (Supplementary Table S1).

**TABLE 1:** Hospitals participating in this trial.

Code	Participating hospitals	Expected recruitment number
1	Beijing Children’s Hospital, Capital Medical University	14
2	Children’s Hospital of Hebei Province	36
3	Baoding Children’s Hospital	30
4	Kunming Children’s Hospital	24

**TABLE 2:** Schedule of enrollment, interventions, and assessments.

Items	Study period (The beginning of meropenem treatment is set as 0 h)			
	Baseline (-3-1 day)	Initial stage (24–72 h)	Termination of meropenem treatment	Follow-up visit (28–32 days after discharge)
Informed consent form (ICF)	√			
Demographic data^a^	√			
Inclusion/exclusion criteria	√			
Randomization and determination of the treatment regimen	√			
Symptoms, signs and physical examination	√	√	√	√
Blood routine^b^	√	√	√	√
CRP and PCT	√	√	√	√
Imaging features of chest X-ray or CT	√	√	√	√
Etiological examination^c^	√	√	√	√
Liver and kidney function^d^ ^,^ ^e^	√	√	√	√
Adverse events		√	√	√
Dose, frequency and time point of meropenem treatment		√	√	
Sampling time of plasma samples		√	√	
Case report form (CRF)	√	√	√	√
Blood drug concentration test		√	√	
Efficacy assessment		√	√	√
Safety assessment		√	√	√

^a^ Demographic data includes age, sex, nation, native place and so on.

^b^ Blood routine tests include red blood cells, white blood cells, platelets, neutrophil percentage, and lymphocyte percentage.

^c^ Etiological examination includes culture, molecular detection, serological and immunological detection.

^d^ Liver function tests include alanine and aspartate aminotransferases, alkaline phosphatase level, and total bilirubin.

^e^ Kidney function tests include blood creatinine and blood urea nitrogen.

### Ethics and information dissemination

The protocol for this RCT has been approved by the ethics committee of Beijing Children’s Hospital ([2022]-E-133-Y). This trial has been registered in the Chinese Clinical Trial Registry (ChiCTR2200061207) and will be conducted in accordance with the Declaration of Helsinki 1964 and its later amendments and Good Clinical Practice guidelines. For each significant change to the protocol, an amendment must be approved by the ethics committee. Written informed consent form (ICF) will be required before recruit tment. The guardians of participants must sign the ICF, moreover, the participants aged 8–15 years must sign the ICF personally. The results of this RCT will be disseminated to the public through relevant academic journals/conferences/workshops.

### Diagnostic criteria of severe pneumonia

The diagnostic criteria for severe pneumonia are based on the Pneumonia Severity Index (PSI) (Supplementary Table S2) (Fine et al., 1997) and Diagnosis and Treatment Guidelines for Community-acquired Pneumonia in Children (2019 edition) (National Health Commission and Medicine, 2019).

The diagnostic criteria of pneumonia in children are:

1) symptoms of respiratory-tract infection (e.g., fever and cough);
2) signs of lung infection, such as low breath sounds, dry and wet rales;
3) results of imaging examination suggest abnormal changes in lung parenchyma or interstitium.

Patients with any of the following condition are diagnosed as having severe pneumonia:

1) poor physical status (pale or gray complexion and poor response to the surrounding environment);
2) consciousness disorders;
3) the presence of one of the following symptoms of hypoxemia: cyanosis, accelerated respiration, respiratory rate ≥70 beats/min (infant), respiratory rate ≥50 beats/min (>1 year old), assisted breathing (moan, nasal fan, three concave sign), intermittent apnea, oxygen saturation <92%;
4) ultra-high fever (>41°C) or continuous high fever (>39°C) for >5 days;
5) dehydration or antifeeding;
6) radiograph or computed tomography of the chest shows ≥2/3 lung infiltration, multilobed lung infiltration, pleural effusion, pneumothorax, atelectasis, lung necrosis, or lung abscess;
7) systemic complications.

### Inclusion criteria

Patients who meet the criteria shown below may be enrolled in this RCT:

1) children aged from 3 months to 15 years;
2) pathogen-based or clinical diagnosis of severe pneumonia;
3) suitable for meropenem treatment and the duration of meropenem treatment is estimated to be more than 48 h;
4) normal renal function (considering urinary protein, urinary red blood cells, serum creatinine, blood urea nitrogen, and creatinine clearance rate) (Lameire et al., 2021)
5) ICF signed by the parent or legal guardian of the children. Children aged from 8 years to 15 years must sign the ICF personally.

### Exclusion criteria

Patients who meet any of the following exclusion criteria will not be admitted to this RCT:

1) allergic to meropenem;
2) participating in other clinical trials or receiving other antibiotics with a similar antibacterial spectrum to meropenem (including third generation cephalosporins, other carbapenem antibiotics and so on) or receiving hormones;
3) predicted survival is shorter than treatment duration
4) receiving renal dialysis therapy or extracorporeal membrane oxygenation;
5) having other factors that clinicians consider unsuitable for RCT inclusion.

### Recruitment strategies

We will recruit hospitalized children from Beijing Children’s Hospital, Children’s Hospital of Hebei Province, Baoding Children’s Hospital and Kunming Children’s Hospital. Advertisements for recruitment on social media (e.g., WeChat™ and QQ™) will be employed. The clinicians of these four hospitals will be in charge of recruitment of patients with severe pneumonia.

### Randomization and blinding

To minimize the risk of a selection bias, we have authorized Beijing Liuyuan Spatial Information Technology (http://h6world.cn/) to carry out block randomization for enrolled patients using a computer-based random-number generator. The size of the randomization blocks is four. Participants will be assigned randomly into a conventional treatment group and model treatment group at a 1:1 ratio.

The randomized code (derived from the randomization system of Beijing Liuyuan Spatial Information Technology) will be the unique identification code for participants. The only individuals who are blinded to the treatment regimen are those investigators involved in data analysis, i.e., laboratory quantitation and statistical analysis.

### Intervention

After enrollment, patients will be assigned randomly to the conventional treatment group (meropenem: 20 mg/kg, q8 h, with 0.5–1 h infusion) or model treatment group (meropenem: 20 mg/kg, q8 h, with 4 h infusion). The steady-state plasma concentration of meropenem will be reached after 24 h of dosing. Hence, an opportunistic method for blood sampling will be used to collect two plasma samples for each patient to measure the drug concentration 24 h after initial treatment.

### Primary outcome

The primary outcome will be 70% fT > MIC (The time during which the free plasma concentration of meropenem exceeds the MIC is 70% of the dosing interval). We will calculate the 70% fT > MIC for each patient and then compare rates of 70% fT > MIC between two groups. Meropenem is a time-dependent drug, and its efficacy is dependent upon the percentage of time that its plasma concentration exceeds the MIC. The antibacterial activity of meropenem occurs when fT > MIC is more than 40% of the dosing interval, but a higher level (70%–80%) is required in critically ill patients (Cies et al., 2017; Heil et al., 2018).

### Secondary outcomes

There are five secondary outcomes in this RCT: 1) prevalence of meropenem therapy failure: the symptoms and laboratory infection indicators persist, worsen or relapse. Moreover, we consider the following conditions to be meropenem treatment failure, such as increasing the meropenem dose, adding other antibiotics to meropenem treatment, stopping meropenem treatment and switching to other antibiotics, and rehospitalization (Torres et al., 2018).

2) duration of meropenem treatment: meropenem will be generally used until 72–96 h after patient’s temperature is normal and the symptoms subsided (National Health Commission, 2015);
3) changes in levels of inflammatory indicators, including the counts of white blood cells and neutrophils, as well as levels of C-reactive protein (CRP) and procalcitonin (PCT). The normal values of the above laboratory tests are shown in Supplementary Table S3
4) changes in imaging examination results (e.g., radiography or computed tomography of the chest): changes will be jointly assessed by the radiologist and the physician in charge;
5) prevalence of adverse events (AEs): diarrhea, hypokalaemia, anaemia, constipation, nausea, vomiting, rash, granulocytopenia, liver/kidney dysfunction, and so on (Torres et al., 2018).

### Calculation of sample size

According to our previous study, 70% fT > MIC of the model treatment group was 73.5%, whereas 70% fT > MIC of the convention al treatment group was 11.4% with MIC = 1 mg/L (Wang et al., 2020). Then, we set *α* = 0.05 and *β* = 0.2, and the ratio of two groups was 1:1. PASS 15.0 (NCSS, LLC, Kaysville, UT, United States) was used to calculate the sample size. It was concluded that each group required 45 patients. Considering a dropout rate of 10%, we will enroll 50 participants for each treatment group.

### Data management and statistical analyses

There will be three main sources of raw data for this RCT: 1) demographic information, diagnosis, clinical data, and laboratory data can be traced to the medical-record system of each branch center; 2) information on treatment and sampling should be documented clearly by clinicians; 3) the drug concentration in plasma should be recorded by the testing personnel. The information stated above will be entered into an electronic case report form provided by Beijing Liuyuan Spatial Information Technology. To ensure the authenticity and accuracy of data, we will assign two personnel to enter information in parallel, which will then be checked again by other personnel.

We will use NONMEN 7.2.0 (Icon Development Solutions, Dublin. Ireland) to calculate the time when fT > MIC for each patient to determine whether it reached 70% of the dosing interval, and then we will compare control rates between two groups. Subsequently, we will assign a statistician to conduct statistical analyses using SPSS 23.0 (IBM, Armonk, NY, United States). The specific statistical methods we will use are as follows:

First, we will describe the data in the demographic information and outcome indicators. For quantitative data, we will calculate values of the mean ± standard deviation or medians and quartiles according to their distribution characteristics. For qualitative data, we will calculate the ratio or rate. Next, we will conduct a univariate analysis of baseline data and outcome indicators. For quantitative data, we will carry out the Student’s t-test, ANOVA, or rank-sum test. For qualitative data, we will carry out the chi-square test. *p* < 0.05 (two-sided) will be considered significant.

### Safety monitoring

Meropenem is a key antibiotic for the treatment of severe pneumonia. The dose and frequency of meropenem will not be changed in this RCT: we will only extend the duration of intravenous infusion. Hence, the probability of AEs will be low. Nevertheless, if moderate to severe AEs occur, we will stop the study immediately and provide effective treatment (Supplementary Tables S4,S5). All kinds of AEs will be reported to the project leader and ethics committee.

### Quality control

We will convene several mobilization meetings for project implementation with each branch center to train clinicians on the details of the sampling and collection of data before RCT initiation. During RCT implementation, the principal in each branch center will ensure the standard of sampling and authenticity of data collection. To strengthen management, the project leader will check the collection of samples and clinical data regularly.

## Discussion

Based on a PPK study of meropenem in children, we aim (for the first time) to conduct a multicenter RCT and compare the 70% fT > MIC, efficacy, and safety between a conventional regimen and model regimen for meropenem against severe pneumonia. Our RCT will provide PD data to optimize meropenem use in treatment of severe pneumonia.

Pneumonia is a serious acute respiratory infection that affects the alveoli and distal bronchial tree of the lungs (Torres et al., 2021). The main clinical characteristics of pneumonia are fever, cough, dyspnea, and medium/fine rales in the lungs. If the disease progresses to severe pneumonia, patients may develop local and systemic complications such as pleural effusion, empyema, bacteremia, sepsis, septic shock or multiple-organ failure (de Benedictis et al., 2020). In China, the most common pathogenic bacteria causing pneumonia are *S. pneumoniae, H. influenzae*, and *Klebsiella pneumoniae* (Li et al., 2021). *β*-Lactam antibiotics are mainly used in clinical treatment according to etiological results or regional epidemiological situation (National Health Commission and Medicine, 2019). However, in recent years, the resistance of pathogenic bacteria to antibiotics has become a key problem in clinical treatment. Clinical application of antibiotics is based mainly on drug instructions/guidelines. The drug concentration in the plasma of some children cannot reach the effective therapeutic concentration due to PK differences, which is an important reason for treatment failure and drug resistance. Differences in PK parameters in children are even more important than those in adults (Kearns et al., 2003). Thus, clarifying the rational use of antibiotics with PK and PD studies not only helps to improve the treatment effect, but also plays an important part in preventing bacterial resistance to drugs.

Meropenem is a second-generation carbapenem which has strong activity against Gram-negative and Gram-positive bacteria by inhibiting the development of bacterial cell walls by targeting penicillin-binding proteins (Baldwin et al., 2008). It is used when children have sepsis or septic shock, or when there is a high degree of clinical suspicion of Gram-negative, extended spectyum *β* lactamase (ESBLs)-producing bacterial infection. In severe pneumonia, if the aforementioned situations occur, meropenem is also applied in anti-infective therapy (National Health Commission and Medicine, 2019). For severe pneumonia, the treatment regimen of meropenem is recommended to be 20 mg/kg, q8h, with 0.5–1 h infusion. However, when we measured the meropenem concentration in plasma and therapeutic effect in children with severe bacterial infection, only 11.4% of children receiving conventional treatment achieved the target goal of 70% fT > MIC (MIC = 1 mg/L) (Wang et al., 2020). Moreover, the cure rate of children with the target goal (92.86%) was significantly higher than that of the children without the target goal (64.1%) (Wang et al., 2022). In addition, studies have shown that EI of meropenem will significantly improve the clinical rate of patients (Lorente et al., 2006). According to data from the China Antimicrobial Surveillance Network (CHINET) and the study of Peng and colleagues, the prevalence of drug resistance of *K. pneumoniae* and *S. pneumoniae* to meropenem is 24.2% and 10%, respectively (Peng et al., 2021; Network, 2022). To avoid the treatment failure and drug resistance caused by the drug concentration in blood not reaching the target goal, carrying out research on PK and PD for meropenem treatment is crucial.

To ensure that the findings of this RCT can systematically reflect the PD characteristics of meropenem in treatment of severe pneumonia, 70% fT > MIC will be employed as the indicator of the primary outcome. Due to the low prevalence of positivity of bacterial cultures in children, population-based epidemiological data will be used to decide the MIC. According to the bacterial-susceptibility data for meropenem, the MICs of *S. pneumoniae* and *H. influenzae* is 0.25–0.5 mg/L (CLSI, 2019), so we set MIC = 1 mg/L as the parameter for calculation of 70% fT > MIC. For indicators of secondary outcomes, we will use the major indicators employed for evaluation of the efficacy and safety for antibiotics: prevalence of meropenem treatment failure, duration of antibiotic therapy, changes in levels of inflammatory indicators, changes in imaging findings, and prevalence of AEs (Molyneux et al., 2011; Stocker et al., 2017).

There are two main limitations in our RCT. First, the prevalence of positive bacterial cultures in children with severe pneumonia is low. Hence, we have selected nationwide epidemiological data from the Chinese Center for Disease Control and Prevention, and set the MIC as 1 mg/ml to determine 70% fT > MIC. Secondly, although creatinine was found to be a significant factor affecting the PK parameters of meropenem in our previous PPK study, only children with normal renal function will be included in this study due to safety considerations. Hence, in the future, another PD study should be carried out in children with abnormal renal function to optimize the drug-administration regimen for this population.

In conclusion, this RCT of meropenem use in treatment of severe pneumonia in children can provide a basis for promoting rational application of antibiotics and improving the therapeutic effect of severe pneumonia in children. Leroux et al., 2016. Zhao et al., 2013.

## Data Availability

The original contributions presented in the study are included in the article/Supplementary Material, further inquiries can be directed to the corresponding authors.
