# Supplementary material for: Meropenem for children with severe pneumonia: Protocol for a randomized controlled trial
Source: Front Pharmacol. 2022 Nov 17;13:1021661. doi: 10.3389/fphar.2022.1021661 (PMC9714324; doi:10.3389/fphar.2022.1021661)
Supplement: Supplementary file 1 [file DataSheet1.docx]

| **Supplementary Table S1｜**CONSORT checklist | | |
| --- | --- | --- |
| Section | Item NO | Checklist item |
| Title and abstract | 1a | Identification as a randomized trial in the title |
|  | 1b | Structured summary of trial design, methods, results, and conclusions |
| Introduction | | |
| Background and objectives | 2a | Scientific background and explanation of rationale |
|  | 2b | Specific objectives or hypotheses |
| Methods | | |
| Trial design | 3a | Description of trial design (such as parallel, factorial) including allocation ratio |
|  | 3b | Important changes to methods after trial commencement, with reasons |
| Participants | 4a | Eligibility criteria for participants |
|  | 4b | Settings and locations where the data were collected |
| Interventions | 5 | The interventions for each group with sufficient details to allow replication, including how and when they were actually administered |
| Outcomes | 6a | Completely defined pre-specified primary and secondary outcome measures, including how and when they were assessed |
|  | 6b | Any changes to trial outcomes after the trial commenced, with reasons |
| Sample size | 7a | How sample size was determined |
|  | 7b | When applicable, explanation of any interim analyses and stopping guidelines |
| Randomization | | |
| Sequence generation | 8a | Method used to generate the random allocation sequence |
|  | 8b | Type of randomization; details of any restriction (such as blocking and block size) |
| Allocation concealment mechanism | 9 | Mechanism used to implement the random allocation sequence (such as sequentially numbered containers), describing any steps taken to conceal the sequence until interventions were assigned |
| Implementation | 10 | Who generated the random allocation sequence, who enrolled participants, and who assigned participants to interventions |
| Blinding | 11a | If done, who was blinded after assignment to interventions (for example, participants, care providers, those assessing outcomes) and how |
|  | 11b | If relevant, description of the similarity of interventions |
| Statistical methods | 12a | Statistical methods used to compare groups for primary and secondary outcomes |
|  | 12b | Methods for additional analyses, such as subgroup analyses and adjusted analyses |
| Results | | |
| Participant flow | 13a | For each group, the numbers of participants who were randomly assigned, received intended treatment, and were analyzed for the primary outcome |
|  | 13b | For each group, losses and exclusions after randomization, together with reasons |
| Recruitment | 14a | Dates defining the periods of recruitment and follow-up |
|  | 14b |  |
| Baseline data | 15 | A table showing baseline demographic and clinical characteristics for each group |
| Numbers analysed | 16 | For each group, number of participants (denominator) included in each analysis and whether the analysis was by original assigned groups |
| Outcomes and estimation | 17a | For each primary and secondary outcome, results for each group, and the estimated effect size and its precision (such as 95% confidence interval) |
|  | 17b |  |
| Ancillary analyses | 18 | Results of any other analyses performed, including subgroup analyses and adjusted analyses, distinguishing pre-specified from exploratory |
| Harms | 19 | All important harms or unintended effects in each group |
| Discussion | | |
| Limitations | 20 | Trial limitations, addressing sources of potential bias, imprecision, and, if relevant, multiplicity of analyses |
| Generalizability | 21 | Generalizability (external validity, applicability) of the trial findings |
| Interpretation | 22 | Interpretation consistent with results, balancing benefits and harms, and considering other relevant evidence |
| Other information | | |
| Registration | 23 | Registration number and name of trial registry |
| Protocol | 24 | Where the full trial protocol can be accessed, if available |
| Funding | 25 | Sources of funding and other support (such as supply of drugs), role of funders |

| **Supplementary Table S2｜**PSI rating scale | |
| --- | --- |
| Indicators | Score |
| Sex |  |
| Male | 0 |
| Female | -10 |
| Residence |  |
| Nursing Home | +10 |
| Combined disease |  |
| Tumor | +30 |
| Hepatopathy | +20 |
| Congestive heart failure | +10 |
| Cerebrovascular disease | +10 |
| Nephropathy | +10 |
| Chest radiography |  |
| Hydrothorax | +10 |
| Signs |  |
| Altered mental status | +20 |
| Respiratory rate ≥30 beats/min | +20 |
| SBP<90mmHg | +20 |
| Body temperature<35℃ or >40℃ | +15 |
| Pulse≥125 beats/min | +10 |
| Laboratory testing |  |
| Blood urea nitrogen ≥11mmol/L | +20 |
| Serum sodium<130mmol/L | +20 |
| Blood glucose≥14mmol/L | +10 |
| Hematocrit value<0.3 | +10 |
| pH<7.35 | +30 |
| PaO_2_<60mmHg or SaO_2_<90% | +10 |

^*^ I:0, II:≤70, III: 71-90, IV: 91-130, V: >130

| **Supplementary Table S3∣**The normal values of laboratory tests | | | | |
| --- | --- | --- | --- | --- |
| Items | Units | Age | Male | Female |
| Red blood cells | ×10^12^/L | 3m-6m | 3.3-5.2 | |
|  |  | 6m-6y | 4.0-5.5 | |
|  |  | 6y-13y | 4.2-5.7 | |
|  |  | 13y-15y | 4.5-5.9 | 4.1-5.3 |
| White blood cells | ×10^9^/L | 3m-6m | 4.3-14.2 | |
|  |  | 6m-1y | 4.8-14.6 | |
|  |  | 1y-2y | 5.1-14.1 | |
|  |  | 2y-6y | 4.4-11.9 | |
|  |  | 6y-13y | 4.3-11.3 | |
|  |  | 13y-15y | 4.1-11.0 | |
| Platelets | ×10^9^/L | 3m-6m | 183-614 | |
|  |  | 6m-1y | 190-579 | |
|  |  | 1y-2y | 190-524 | |
|  |  | 2y-6y | 188-472 | |
|  |  | 6y-12y | 167-453 | |
|  |  | 12y-15y | 150-407 | |
| Neutrophil percentage | % | 3m-6m | 7-56 | |
|  |  | 6m-1y | 9-57 | |
|  |  | 1y-2y | 13-55 | |
|  |  | 2y-6y | 22-65 | |
|  |  | 6y-13y | 31-70 | |
|  |  | 13y-15y | 37-77 | |
| Lymphocyte percentage | % | 3m-6m | 26-83 | |
|  |  | 6m-1y | 31-81 | |
|  |  | 1y-2y | 33-77 | |
|  |  | 2y-6y | 23-69 | |
|  |  | 6y-13y | 23-59 | |
|  |  | 13y-15y | 17-54 | |
| CRP | mg/L | 3m-15y | <8 | |
| PCT | ng/mL | 3m-15y | <0.25 | |
| Alanine aminotransferase | U/L | 3m-1y | 8-71 | |
|  |  | 1y-2y | 8-42 | |
|  |  | 2y-13y | 7-30 | |
|  |  | 13y-15y | 7-43 | 6-29 |
| Aspartate aminotransferase | U/L | 3m-1y | 21-80 | |
|  |  | 1y-2y | 22-59 | |
|  |  | 2y-13y | 14-44 | |
|  |  | 13y-15y | 12-37 | 10-31 |
| Alkaline phosphatase | U/L | 3m-6m | 98-532 | |
|  |  | 6m-1y | 106-420 | |
|  |  | 1y-2y | 128-432 | |
|  |  | 2y-9y | 143-406 | |
|  |  | 9y-12y | 146-500 | |
|  |  | 12y-14y | 160-610 | 81-454 |
|  |  | 14y-15y | 82-603 | 63-327 |
| Total bilirubin | μmol/L | 3m-15y | 3.42-20.5 | |
| Blood creatinine | μmol/L | 3m-2y | 13-33 |  |
|  |  | 2y-6y | 19-44 |  |
|  |  | 6y-13y | 27-66 |  |
|  |  | 13y-15y | 37-93 | 33-75 |
| Blood urea nitrogen | mmol/L | 3m-6m | 0.8-5.3 |  |
|  |  | 6m-1y | 1.1-5.9 |  |
|  |  | 1y-2y | 2.3-6.7 |  |
|  |  | 2y-15y | 2.7-7.0 | 2.5-6.5 |
| Creatinine clearance | ml/min/1.73m^2^ | 3m-15y | 80-130 | |
| Urinary protein |  |  | negative | |
| Urinary red blood cells |  |  | negative | |
| *According to Health Industry Standards of the People's Republic of China (WS/T 779—2021, WS/T 780—2021) | | | | |

| **Supplementary Table S4∣** Classification and management of AEs | | |
| --- | --- | --- |
| Classification | Characteristic | Management |
| Mild | AEs are mild and do not require medical intervention | Keep on study |
| Moderate | AEs are mild and tolerable to the patient but require medical intervention | Stop study |
| Severe | AEs are severe and intolerable to the patient and require medical intervention | Stop study |

| **Supplementary Table S5∣**The judgment criteria for the correlation between AEs and meropenem | |
| --- | --- |
| Correlation | Description |
| Certain | - A clinical event, including a laboratory test abnormality, that occurs in a plausible time relation to drug administration, and which cannot be explained by concurrent disease or other drugs or chemicals. - The response to withdrawal of the drug (dechallenge) should be clinically plausible. - The event must be definitive pharmacologically or phenomenologically, using a satisfactory rechallenge procedure if necessary. |
| Probable/Likely | - A clinical event, including a laboratory test abnormality, with a reasonable time relation to administration of the drug, unlikely to be attributed to concurrent disease or other drugs or chemicals, and which follows a clinically reasonable response on withdrawal (dechallenge). - Rechallenge information is not required to fulfil this definition. |
| Possible | - A clinical event, including a laboratory test abnormality, with a reasonable time relation to administration of the drug, but which could also be explained by concurrent disease or other drugs or chemicals. - Information on drug withdrawal may be lacking or unclear |
| Unlikely | - A clinical event, including a laboratory test abnormality, with a temporal relation to administration of the drug, which makes a causal relation improbable, and in which other drugs, chemicals, or underlying disease provide plausible explanations. |
| Conditional/Unclassified | - A clinical event, including a laboratory test abnormality, reported as an adverse reaction, about which more data are essential for a proper assessment or the additional data are being examined |
| Unassessable/ Unclassifiable | - A report suggesting an adverse reaction that cannot be judged, because information is insufficient or contradictory and cannot be supplemented or verified |
